# Challenges for dermatologists during the COVID-19 pandemic: A qualitative study

**DOI:** 10.1097/JW9.0000000000000013

**Published:** 2022-03-25

**Authors:** Matthew F. Helm, Alexa B. Kimball, Melissa Butt, Heather Stuckey, Heather Costigan, Kanade Shinkai, Arielle R. Nagler

**Affiliations:** a Department of Dermatology, Penn State Hershey, Hershey, Pennsylvania; b Department of Dermatology, Beth Israel Deaconess Medical Center, Harvard Medical School, Boston, Massachusetts; c Department of Internal Medicine, The Penn State Hershey, Hershey, Pennsylvania; d Department of Dermatology, The University of California San Francisco, San Francisco, California; e The Ronald O. Perelman Department of Dermatology, NYU Grossman School of Medicine, New York, New York

**Keywords:** Burnout, COVID-19, work-life integration, stressors, teledermatology, electronic medical record

## Abstract

Burnout is increasing in all fields of medicine, including dermatology. The coronavirus disease 2019 (COVID-19) pandemic presented new and additional challenges for dermatologists.

**Objective:**

Dermatologists of different ages, areas of expertise, and practice settings were convened in 5 focus group to describe the impact of the COVID-19 pandemic on their clinical practice, working environment, and personal lives.

**Methods:**

Qualitative analysis of the discussions w\s performed on the result of the 5 focus groups of dermatologists (n = 22). Groups were prompted with questions relating to their jobs, personal lives, teledermatology, and pandemic. Responses were recorded, transcribed, deidentified, and coded for recurring themes. The focus groups occurred via a secure videoconferencing platform between December 2020 and January 2021. All participants were currently practicing dermatology in a variety of setting including academic institutions, private practices, and multiple practice types. General dermatologists, residents in training, dermatologic surgeons, dermatopathologists, and dermatologists with significant administrative or educational duties were included.

**Results:**

We identified 4 main themes from the focus group discussions regarding dermatologist and physician wellbeing during the COVID-19 pandemic: (1) adjusting to new administrative, staffing, and educational demands; (2) integration of work as a dermatologist with family life; (3) new technologies such as teledermatology; and (4) adjusting to change with redefining personal and professional priorities.

**Limitations:**

The small number of participants in our convenience cohort disproportionately represented academic dermatologists. Impacts of regional COVID-19 vaccination rates and ideological differences in different geographical locations were not assessed. All of our participants were located in the United States. Physicians severely impacted by health or financial concerns may not have been able to participate in our study. We did not have a comparison group and did not measure or assess burnout in individual participants.

**Conclusion:**

During the COVID-19 pandemic, there were common changes and stressors that dermatologists experienced, which affected physician wellbeing. Identifying and addressing these changes could offer the opportunity to improve the wellbeing of dermatologists.

**Highlights:**

- Four major stressors were identified across dermatologists’ experiences during the COVID-19 pandemic: (1) adjusting to new administrative, staffing, and educational demands; (2) integration of work as a dermatologist with family life; (3) rapidly pivoting to new technologies such as teledermatology; and (4) adjusting to change with redefining personal and professional priorities.
- Identifying stressors may provide opportunities to implement interventions against burnout in dermatology.

**What is known about this subject in regard to women and their families?**

- Burnout has been increasing among female dermatologists

**What is new from this article as messages for women and their families?**

- Rapid changes to the physical workplace, patient care scheduling, child and elder care, and different administrative burdens during the COVID-19 pandemic have placed dermatologists under great stress.
- Identifying stressors and redefining personal and professional priorities provide opportunities to develop mitigating strategies.

## Introduction

Burnout is common among all physicians, including dermatologists. The Medscape National Physician Burnout & Suicide Report in 2021 surveyed 12,000 physicians in over 29 specialties and noted that 42% of all physicians and 36% of dermatologists reported burnout.^13^ Major contributors to burnout include bureaucratic tasks, too many hours at work, electronic medical records, as well as “lack of respect, control, and autonomy.”^4,13^ Other studies have shown that the proportion of physicians with burnout is >40%, compared with 28% in the general US working population.^7^ Physicians have also been shown to have lower satisfaction of work-life integration when compared with the general working population.^23^

Burnout erodes physician wellbeing and leads to increased medical errors and lower patient satisfaction.^19^ Our group has previously proposed burnout mitigation strategies for dermatologists that include the following: (1) focus on incremental changes that help restore autonomy and control over work, (2) connect with colleagues within the dermatology medical community, (3) develop self-awareness and recognition of a perfectionist mindset, and (4) restore meaning to patient care.^16^

The COVID-19 pandemic has imposed additional challenges and stresses to the practice of medicine and has exacerbated issues of social justice and autonomy.^15^ Studies have shown that early in the pandemic, there was a large decrease in average weekly visits in dermatology, leading to gaps in patient care and revenue losses.^11,12^ This study is a qualitative analysis of interviews with dermatologists about their experiences during the COVID-19 pandemic to identify key stressors and opportunities to alleviate burnout.

## Methods

This qualitative study included a convenience sample comprising 5 focus groups (n = 22) of board-certified dermatologists and dermatology residents. Participants engaged in semistructured interviews regarding the impact of COVID-19 on their clinical work and education. Focus groups were conducted remotely via ZOOM and moderated by a single research interviewer. Focus group participants included general dermatologists, dermatologic surgeons, dermatopathologists, dermatology residents, as well as subspecialty dermatologists. An effort was made to include physicians from different age groups, gender, ethnic groups, and geographic locations. All focus group sessions were audiorecorded for verbatim transcription by a professional transcription agency. Fifteen questions were developed to prompt discussion (see Supplement for Interview Guide http://links.lww.com/IJWD/A1), and participants were encouraged to share their experiences and viewpoints beyond the questions. The number of focus groups conducted was determined at the point when no additional new categories or important information were identified in the data.

**Fig. 1.**
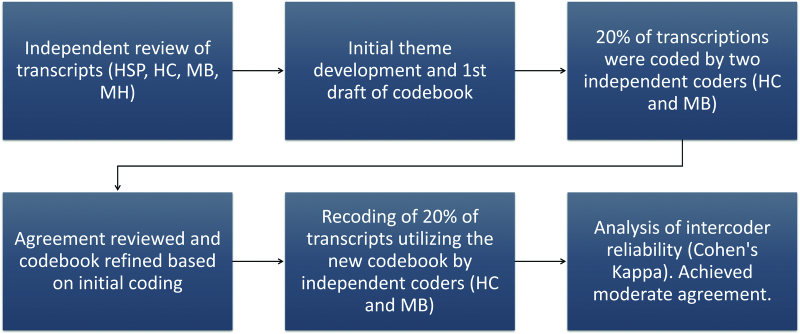
Process for determining the codebook.

Transcripts were deidentified and reviewed for accuracy. For thematic analysis, an initial codebook was derived based on a review of all transcripts using an inductive, emergent approach (HC, HSP, MB, and MFH). The codebook was then applied to approximately 20% of the focus group content to establish interrater reliability using Cohen’s Kappa and to refine the codebook.^17^ After the first review, minor changes were made to restructure the codebook into a final version (Fig. 1). Two separate coders (MB and HC) coded 20% of the material to demonstrate substantial agreement. After the substantial agreement was reached (Kappa = 0.66), a single coder (HC) then coded the remaining 4 focus groups. After completion of the coding, references were reviewed and finalized to establish themes and a concept map. Descriptive hematic analysis was utilized to string together coded pieces of data into themes that emerged in importance from the coded focus group data.^5^ All qualitative data were coded and analyzed using NVivo Version 12 (QSR International). This research was approved by the Penn State Institutional Review Board.

## Results

**Fig. 2.**
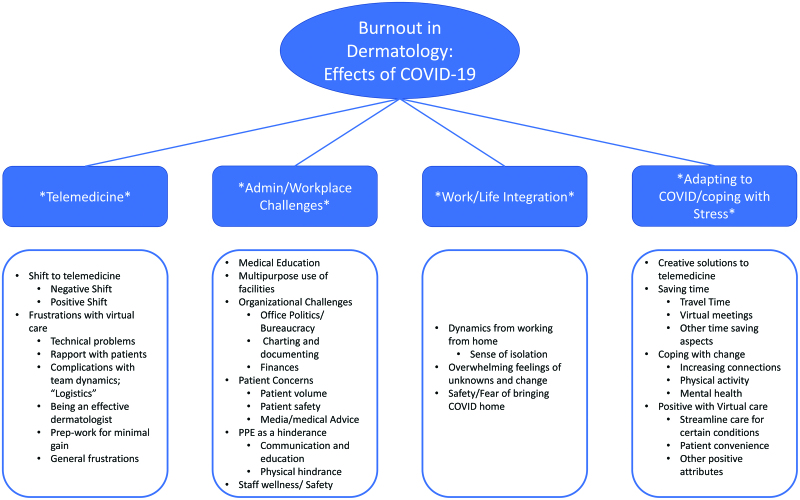
Common challenges that dermatologists experienced during the first year of the COVID-19 pandemic.

Twenty-two dermatologists participated in 5 different focus groups that were conducted from December 2020 to January 2021. Groups were asked demographic questions to determine participants’ practice locations (community based/private practice 27%; university based/academic 68%; multiple practice types 18% [some participants had more than one practice type], gender [male 36%, female 64%], race/ethnicity [white 77%, black 5%, Asian 14%, 2 or more 5%], age, and practice setting [academic, group, private practice]) (Table 1). Data saturation was achieved by the fifth focus group. Review of the coded transcripts revealed 4 common challenges that dermatologists experienced during the first year of the COVID-19 pandemic (Fig. 2): (1) adjustment to workplace, cultural, and administrative changes; (2) integrating work as a dermatologist/life with family; (3) rapid pivoting to new technologies such as teledermatology; and (4) adjustment to change and redefining personal and professional priorities. Examples of quotations from the focus groups that relate to each theme can be found in Table 2.

**Table 1.** Participant characteristics (n = 22)

Sex	N(%)
Male	8 (36)
Female	14 (64)
Race	
White	17 (77)
Black	1 (5)
Asian	3 (14)
Two or more	1 (5)
Years in practice	
Still in training 2 years	5 (23)
3–5 years	3 (14)
6–10 years	7 (32)
11–20 years	3 (14)
21+ years	4 (16)
Practice type	
Community-based/private practice	6 (27)
University based/academic	15 (66)
Multispecialty	4 (16)
Practice focus	
General dermatology	18 (82)
Dermatopathology	6 (27)
Dermatologic surgery	9 (41)
Cosmetic dermatology	8 (36)
Pediatric dermatology	5 (23)
Clinical care percentage (%)	
0–40	2 (9)
41–60	3 (14)
61–80	3 (14)
81–100	14 (64)
Works with trainees	
Yes	16 (73)
No	6 (27)

**Table 2.** Representative quotations

Challenge 1: Adjusting to workplace, cultural and administrative changes	
Administrative burden	“And so, as you can imagine, being still a relatively high-volume provider, I suddenly had a lot more charting and administrative things that don’t have to do with high level decision making, that I’ve had to do on my own because we didn’t have the manpower that normally works with me.”
Administrative burden	“I think in those initial first few months for us, I would say from March until June, July, there’s a lot more administrative work as we canceled and rescheduled and tried to triage patients.”
Medical education	I have had the worry about keeping the education going for all of the residents and the students who rotate with us. And it has been quite a rollercoaster.
Medical education	I feel for the students. There are a lot of interns graduating this year who have lost a significant amount of their clinical time.
Team changes	“And also, I wasn’t allowed to have a medical scribe anymore, which was something that really had decreased the physician burnout, the workload and burnout, at least at our hospital, and had really been valued by physicians. But when we had to minimize the number of people in clinic, and cut costs, that was the first thing that went.”
Redeployment/new responsibilities	Our department was I guess redeployed… not deployed clinically, but redeployed in COVID efforts, 150% of my time was spent doing those 7 days a week. We covered COVID callbacks for all patients, contact tracing, working with the department of health, et cetera.
Redeployment/new responsibilities	“And the hospital may call me or my sister up to actually go in, and be general medicine docs, if they run out of docs, because a lot of the staff in the hospital are now COVID positive and there’s a shortage of staff. So that’s been really quite stressful.”
Financial stress	Our biggest headaches were just trying to navigate a lot of the ways that we could keep our staff employed because we made a commitment to them that we weren’t going to lay off anyone.
Financial stress	My group did get to take advantage of the Paycheck Protection Program, and we met all the qualifications, so we were given our loan. But that required quite a bit of administrative work to make sure that we met all of the criteria there.
Staff wellness and safety	I also was super worried about my staff that I really love and care for like family… my first priority was to keep them safe. I guess I worry a lot about our residents, because I feel like they’re a population where a lot of them have been removed from their families and loved ones.
PPE as a hindrance	[I felt] isolated from my patients by plastic and cloth, and PPE, and we’re isolated socially because we’re trying to be good stewards and role models…. It’s been a tough year.
**Challenge 2: Integrating work as a dermatologist/life with family**	
Dynamics juggling work and life duties	We’re all used to being busy and juggling lots of things, but the sheer volume that’s come all at once, it’s kind of like drinking from a fire hydrant.
Dynamics juggling work and life duties	There are 3 kids at home who are doing school from home, and managing that, this has just been a wretched year.
Changing circumstances	I remember waking up and being like… we’d play hot potato with our children.
Changing circumstances	I think there’s even more of an expectation that meetings are going to happen on weekends and late at night. And I feel like they never end.
Fear of spreading COVID	Scared for my patients, my family, myself, with everything kind of going on, and my colleagues as we heard about these cities like New York, Seattle, Louisiana, and now it’s just everywhere. I think that is still present, you’re just worried.
Fear of spreading COVID	I think there was definitely some anxiety. Not that’s why for me directly but relatives knowing that I was going to work and still seeing patients even though we weren’t seeing as many patients, it was more emergencies back then.
**Challenge 3: Rapidly pivoting to new technologies such as teledermatology**	
Effective dermatologist	Dermatology is a very visual field. I wasn’t able to be an effective dermatologist. And then a lot of times when it was a spot that I had to look at, I ended up just having to tell them they had to come in anyways so I thought it was redundant for myself and for the patient.
Complications with team resources	And of course, the administration of rescheduling patients and seeing what would be appropriate for virtual visits and not... I mean, it was just a mountain of work.
Preparatory work for minimal gains	I just think it took maybe in many cases, more time, sometimes even double the amount of time for the patient. And then as an attending, I just think that my patient population has changed a bit for teledermatology.
Rapport with patients	No, I think the formality of the patient physician relationship weakens for sure when you’re suddenly in somebody’s home via teleconference.
Technology issues	Younger patients, it’s great, but for kind of the more elderly population, think it’s a struggle.
Effective dermatologist	So for me, there’s no such thing as virtual dermatopathologist, people have to come in and get a biopsy.
Convenience for patients	That can work really nicely for the schedule and you can do them that service than have them drive so far or whatever. Definitely has its place and it didn’t affect me negatively.
Creative telemedicine alternatives	And the way that we would do it so they feel comfortable is we actually started operating drive-through visits
Negative transition	Yeah. I found telemedicine at the beginning to be very difficult and very slow.
Negative transition	But the very sudden transition to the sort of COVID crisis in the beginning, I think pointed out a lot of flaws or things that we hadn’t anticipated.
**Challenge 4: Adjusting to change and redefining personal/professional priorities**	
Marginalization as a specialty	People were like, “We’re not wasting our PPE on a dermatologist.” And I would basically say, “Look, you guys brought me in to see this I’m being asked to go in, so I feel like I should have PPE for that reason, or don’t call the consult.
Marginalization as a specialty	We’ll just take a picture, and we’ll send it to you.” And what I would get was the photograph from an iPhone 6, through a Ziploc bag, with a glove… And it was like, I couldn’t do anything, and then I would end up basically having to go in anyway. And I think there was a little bit of this sort of perception of, who] is important in the hospital system, and who wasn’t?
Connection with colleagues	And I feel like the conversations at work, people were really checking in. Before it used to be like, “Oh, how’s it going? How are you?” And then we really started having some pretty great conversations at work.
Connection with family	I will say, for my part, it was almost a gift in a way to be able to be home with the kids because I never really had that.
Connection with nature	Nature has been very soothing for me, so I make a point of doing that and it really brings me down in terms of stress levels.
Self-care	I think that was one of the maybe very, very tiny silver linings of all this was kind of realizing what you should make time for, what your priorities should be.
Self-care	You cannot be very helpful to your family if you’re stressed out and not taking care of yourself.
Physical activity for stress relief	So, I never had had a really regular fitness routine, and all of a sudden I found a lot of time on my hands in March. So I started working out regularly, 5 days a week.
Ability to identify and fix problems	We used a lot of the down time in our department to work on templates, do cross training, and look at what was optimal in terms of staffing, and make sure that when we came back, we had maybe a better strategy than we had before.
Streamlining patient care	once the resistance to telemedicine was down, because it basically had to be, we learned there were a lot of things we could have been doing by telemedicine, and the patients learned that there were a lot of things we could be doing by telemedicine.

PPE, personal protective equipment.

### Challenge 1: Adjusting to workplace, cultural, and administrative changes

Workplace concerns identified by participants included patient safety, acquisition of adequate personal protective equipment, continuing medical education, financing, and staffing challenges. Participants were concerned about exposure to patients who were unwilling to follow policies on mask wearing and social distancing. Personal protective equipment also posed challenges in discerning social cues and developing personal connections with patients.

More time was allocated to answering patients’ questions about the pandemic, vaccination, social distancing, and other topics not part of a dermatologist’s typical scope of practice. Many expressed gratitude for the opportunity to help their patients understand and cope with the pandemic and frustration when patients expressed disagreement with medical advice.

Rapidly changing and conflicting local and federal guidelines created barriers to treatments, making it difficult to decide, which tumors required the most urgent management and which ones could be deferred or delayed. Dermatologists whose practices remained opened were forced to make staffing decisions reducing staff hours or laying off staff. Some support staff found unemployment benefits to be comparable to or even to exceed usual wages and were unwilling to return to a workplace considered risky.

Academic dermatologists reported challenges finding innovative ways to continue to teach medical students and residents, although complying with social distancing restrictions. Didactic sessions and clinical rotations rapidly transitioned to virtual learning. Many considered these sessions inadequate substitutes for in person learning experiences.

### Challenge 2: Integrating work as a dermatologist/life with family

The stress of integrating home and work life presented in 3 main ways during the height of the COVID-19 pandemic: (1) working from home while juggling familial responsibilities, (2) feeling overwhelmed about the health risk of coronavirus and protecting family members from becoming ill, and (3) feelings of isolation and loneliness.

Dermatologists in our focus groups with school aged children found it difficult to balance work responsibilities, teach children, and provide emotional support for family members. Daycare and school closures schedules made it difficult for caregivers to develop routines. Several participants expressed frustration about a loss of boundaries between work and home life with meetings occurring late at night or on the weekends. Participants discussed hesitance around completing total body skin examinations because of potential exposure to infectious particles. Fear of contracting COVID at work was particularly prevalent among physicians who were providing intergenerational care in their families (caring for elderly parents and young children).

### Challenge 3: Rapidly pivoting to new technologies such as teledermatology

Practices that had invested in robust electronic medical record systems with telemedicine capabilities were able to rapidly implement new clinical workflows to allow patient care to continue through teledermatology. Many dermatologists were forced to adapt before they were knowledgeable or confident in teledermatology best practices. Poor quality of patient obtained images often precluded confident or meaningful diagnosis. Teledermatology visits and documentation were often more time consuming than in person visits. Technological malfunctions for both the patient and the physician often slowed visits. Mohs surgeons and dermatopathologists were unable to utilize teledermatology because of the procedural nature of their work.

Despite some of these challenges, dermatologists spoke about some of the positive outcomes of the rapid transition and uptake of teledermatology. Patients saved time by participating in teledermatology visits and were able to avoid contact with potentially sick individuals. Access was increased to those who would not be able to have seen a dermatologist otherwise given the circumstances. Dermatologists noted that teledermatology encounters that focused on the effects of isotretinoin therapy, acne, rosacea, or medication refills worked well.

More provider to patient interactions occurred through electronic messaging. Some dermatologists stated that it was a good way to communicate to their patients without risking spread of the virus. Other dermatologists felt that their volume of messages increased substantially and that they were being asked to address completely new issues and diagnoses over a messaging system that did not allow them to bill for their services.

### Challenge 4: Adjusting to change and redefining personal/professional priorities

The COVID-19 pandemic challenged some dermatologists’ sense of purpose. Many felt less important or marginalized as their practices were deemed nonessential and they were asked to close or were reassigned to other duties.

Positive impacts that resulted from the pandemic included participation in virtual national meetings that would otherwise have been inaccessible, saving time by attending workplace meetings from home, and saving time traveling between different practice locations. The pandemic forced some to slow down and remember the key motivators that drew them into medicine in the first place. Extra time usually allocated for a commute was relocated to engage in more outside activities such as walks and hikes. Although some dermatologists in large metropolitan cities were confined to small apartments, others however were able to access nature in ways that were calming and restorative. Some dermatologists purposefully limited intake of news or social media and many dermatologists had newfound gratitude for their support networks.

## Discussion

Our qualitative study identified 4 common challenges encountered by dermatologists during the COVID-19 pandemic. Although some themes and elements were specific to dermatologists, others were generalizable to healthcare workers during the pandemic. Challenges that emerged that were specific to dermatologists included: risks of viral transmission because of prolonged close contact during routine total body skin examinations, marginalization as a specialty, teledermatology, and increased requests to evaluate clinical images leading to increased message burden. Many dermatologists felt their values and core purpose was challenged during COVID-19.^21^

Academic dermatologists and those who were earlier in their career are more likely to utilize teledermatology during the COVID-19 pandemic although many practices did make this switch.^8,12^ Although teledermatology can be an effective, convenient, and cost-effective platform to improve access to care,^24^ many clinicians in our study found interactions to be time consuming and problematic. This sentiment is echoed in patient survey research, which has shown that patients found teledermatology to be an efficient and safe method for quality care through the pandemic^25^ face-to-face visits are often preferred.^6^

Pandemic stressors generalized to other healthcare workers included: physical safety, loss of work, financial stressors, demands of providing high-quality medical education, rapid changes to the physical workplace and patient care scheduling, child and elder care, and different administrative burdens and responsibilities. Rates of infection and death were high among healthcare workers showing the risk and danger of being on the “frontline.”^2^ A recent survey of academic dermatologists indicated that 85% believed that the COVID-19 pandemic contributed to their burnout, with uncertainty about the future, additional duties, and fear of exposing loved ones as the main factors.^22^

Future studies should assess the ongoing impact of the pandemic on dermatologists’ burnout, family life, workplace happiness, and the use of teledermatology. Limitations of our study include the small number of participants in our convenience cohort, the disproportionate representation of academic dermatologists, and the failure to address impacts of regional COVID-19 vaccination rates and ideological differences in different geographical locations. All of our participants were located in the United States. Physicians severely impacted by health or financial concerns may not have been able to participate in our study. We did not have a comparison group and did not measure or assess burnout in individual participants.

## Conclusions

All 4 themes that were highlighted in this qualitative study were a result of changes to professional and personal lives of dermatologists brought about by the COVID-19 pandemic. With these changes, dermatologists were called to adapt and adjust to an uncertain future. Adjustment to change could be an important contributor to burnout that should be investigated further.

## Acknowledgments

We thank our focus group participants for their time and candid answers and the Penn State Qualitative & Mixed Methods Core for expert assistance with transcription and data analysis.

## Conflicts of interest

None.

## Funding

None.

## Study approval

N/A.

## Supplementary Material
