# Supplementary material for: Challenges for dermatologists during the COVID-19 pandemic: A qualitative study
Source: Int J Womens Dermatol. 2022 Mar 25;8(1):e013. doi: 10.1097/JW9.0000000000000013 (PMC9112396; doi:10.1097/JW9.0000000000000013)
Supplement: Supplementary file 1 [file jw9-8-e013-s001.pdf]

DISCLOSURE FORM

Date:______ 2/10221/2
Your Name:__ Alexa B Kimball________________________

Time frame: past 36 months			
2	Grants or contracts from any entity (if not indicated in item #1 above).	Janssen	Fellowship funding
		Abbvie	Fellowship funding
		AbbVie	Investigator
		Bristol Myers Squibb	Investigator
		Anapyts Bio	Investigator
		Incyte	Investigator
		Janssen	Investigator
		Lilly	Investigator
		Novartis	Investigator
		Pfizer	Investigator
		UCB	Investigator
3	Royalties or licenses	Beth Israel Deaconess Medical Center	Licensing of training module for clinical trials to CROs and pharma
4	Consulting fees	AbbVie	
		Bayer	
		Ventyx	
		Moonlake	
		Concert	
		EvoImmune	
		Janssen	
		Lilly	
		Novartis	
		Pfizer	
		UCB	
		Target RWE	
		Incyte	
9	Participation on a Data Safety Monitoring Board or Advisory Board		
		Advisory Committee to Director NIH	unpaid
10	Leadership or fiduciary role in other board, society, committee or advocacy group, paid or unpaid	Beth Israel Lahey Health	BOD Unpaid
		Beth Israel Deaconess Medical Center	BOD Unpaid
		Almirall	BOD
		HS Foundation	BOD
